# Testis specific gene expression drives disease progression and Rituximab resistance in lymphoma

**DOI:** 10.1002/emmm.201303018

**Published:** 2013-08-05

**Authors:** Stefan Knapp

**Affiliations:** Nuffield Department of Clinical Medicine, Target Discovery Institute (TDI) and Structural Genomics Consortium (SGC), University of OxfordOxford, UK

**Keywords:** BET proteins, bromodomains, Cyclon, JQ1, Rituximab

Activation of cancer promoting genes or silencing of tumour suppressors is often due to aberrant expression of transcription factors or transcriptional regulators; interestingly, the expression of many of these transcriptional regulators is usually restricted to male germ cells (Rousseaux et al, 2013; Simpson et al, 2005).

The similarities between germ cell development and cancer have long been noted and resulted in the identification of a number of cancer/testis antigens such as the MAGE family members (Simpson et al, 2005). Although the biological function of many of the cancer/testis antigens is poorly understood, some are known to act as transcriptional regulators. These discoveries spawned the idea that aberrant expression of germ line genes in somatic cells triggers the activation of a gametogenic programme that drives tumourigenesis. A number of these testis/cancer genes comprise epigenetic regulators that play a key role in male genome reprogramming. BRDT, one such regulator, plays a critical function in spermatogenesis and acetylation-dependent chromatin remodelling (Gaucher et al, 2012). Indeed, germ cell and trophoblast differentiation shares many of the phenotypic hallmarks observed in cancer cells including immortalisation, tissue invasion, stimulation of cell migration, induction of angiogenesis and downregulation of the major histocompatibility complex resulting in immune evasion.

In this issue, Emadali et al (2013) report on the identification of the transcriptional regulator CYCLON (cytokine-induced protein with coiled-coil domain) as a downstream effector of oncogenic MYC signalling in lymphoma. CYCLON is highly expressed in testis but expression of this protein is also induced in a hematopoietic cell line by interleukin 3 (IL-3) (Hoshino & Fujii, 2007). The study revealed that in diffuse large B cell lymphoma (DLBCL), the most common form of B-cell non-Hodgkin lymphoma, over-expression of CYCLON mRNA correlated significantly with poor survival. Importantly, CYCLON over-expression was specifically associated with poor survival of lymphoma patients treated with the current standard-of-care chemotherapy CHOP (Cyclophosphamide, Doxorubicin Hydrochloride, Vincristine Sulphate (Oncovin) and Prednisone) in combination with the monoclonal therapeutic antibody Rituximab (Nastoupil et al, 2012) but not in patients treated with chemotherapy alone. This intriguing finding suggests that CYCLON expression confers resistance to Rituximab immunotherapy.

…CYCLON over-expression was specifically associated with poor survival of lymphoma patients treated with the current standard-of-care chemotherapy CHOP (Cyclophosphamide, Doxorubicin Hydrochloride, Vincristine Sulphate (Oncovin) and Prednisone) in combination with the monoclonal therapeutic antibody Rituximab…

The monoclonal antibody Rituximab (trade names Rituxan and MabThera) targets CD20, a surface protein mainly expressed in the B-cell lineage, leading to selective B-cell killing. The Rituximab + CHOP chemotherapy regime is a more efficient treatment strategy compared to chemotherapy alone in high-risk B-cell lymphoma (Michallet et al, 2012). Indeed, Rituximab in combination with CHOP achieves high response rates in B-cell non-Hodgkin lymphoma and has led to significant improvements in overall survival rates. A number of patients, however, develop refractory disease through a mechanism that is poorly understood (Cheson & Leonard, 2008). Studies that identify the mechanisms underlying Rituximab resistance and that lead to additional treatment options are therefore urgently needed.

**Figure 1:**
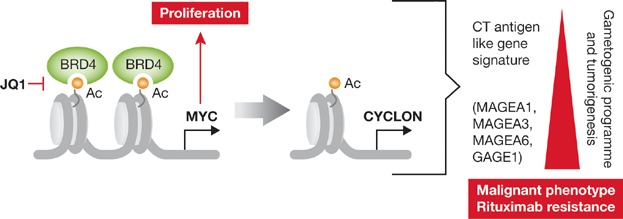
Gene expression events triggering a gametogenic program that leads to Rituximab resistance in lymphoma.

CYCLON knock down in lymphoblastiod cell lines results in the modulation of transcription levels of a specific set of target genes. Interestingly, gene set enrichment analysis (GSEA) correlated with gene signatures of cancer testis antigens of the MAGE family and also matched expression profiles that are characteristic for aggressive subtypes of multiple myeloma. CYCLON knockdown also increased the sensitivity of lymphoma cells to Rituximab *in vitro* and *in vivo*. Importantly, the genetic knockdown effects were mimicked by the BET (bromo and extra terminal) bromodomain inhibitor JQ1 (Filippakopoulos et al, 2010) providing a rational for a new combination therapy in Rituximab resistant lymphoma (Fig 1).

The BET family member BRD4 plays an important role for survival of a number of diverse tumours due to its function promoting transcription of growth promoting and antiapoptotic genes. Dysfunction of BET proteins leads to development of aggressive tumours. For instance, genetic rearrangement resulting in “in frame” fusion of the N-terminal bromodomains of BRD4 and BRD3 with the protein NUT (nuclear protein in testis) give rise to the development of NUT midline carcinoma (NMC), an incurable uniformly fatal subtype of squamous carcinoma and in rare cases also cancers of other origins (French, 2012) providing another example of the strong tumour promoting effect caused by aberrant expression of testis specific proteins. One of the key functions of BRD4 in cancer is to regulate c-Myc expression (Delmore et al, 2011). Indeed, recently developed potent and specific BET inhibitors showed impressive anti-tumour efficacy in animal models and first clinical trials have been initiated recently (http://www.cancer.gov/drugdictionary?cdrid=733799). Since, however, oncogenic MYC is only one factor sensitising cancer cells to BET inhibition and considering its high expression levels in most tumours it is not likely that this oncogene will have predictive value for patient stratification. The study by Emadali et al (2013) makes therefore an important contribution by identifying the nuclear, male germ cell specific factor, CYCLON as a downstream effector of oncogenic MYC signalling in lymphoma. One of the open questions is now how MYC cooperates with CYCLON to give rise to changes in gene expression that lead to Rituximab resistance and disease progression. Further mechanistic studies are therefore needed to elucidate the molecular mechanisms of this regulatory gene expression network and to establish new therapeutic strategies for the treatment of Rituximab resistant lymphoma.

…makes therefore an important contribution by identifying the nuclear, male germ cell specific factor, CYCLON as a downstream effector of oncogenic MYC signalling in lymphoma.

The author declares that he has no conflict of interest.
